# The ethics of research involving biobanking specimens from Kenyan children and adolescents living with HIV: discrepancies between individual perceptions and policy considerations

**DOI:** 10.1186/s12910-026-01421-7

**Published:** 2026-02-26

**Authors:** Kevin Griffee, Winstone Nyandiko, Sakshi Sawarkar, Ashley Chory, Josephine Aluoch, Emma Gillette, Michael Scanlon, Hillary Koros, Daniel Lagat, Nandini Choudhury, Dennis Munyoro, Celestine Ashimosi, Whitney Biegon, Janet Lidweye, Jack Nyagaya, Allison DeLong, Violet Naanyu, Rachel Vreeman, Rami Kantor

**Affiliations:** 1 David Geffen School of Medicine at UCLA, Los Angeles, USA; 2 College of Health Sciences, Moi University, Eldoret, Kenya; 3 Academic Model Providing Access to Healthcare, Eldoret, Kenya; 4 Icahn School of Medicine at Mount Sinai, New York, USA; 5 Center for Global Health, Indiana University, Indianapolis, USA; 6 School of Arts and Social Sciences, Moi University, Eldoret, Kenya; 7 Department of Population Health, NYU Grossman School of Medicine, New York, USA; 8 School of Public Health, Brown University, Providence, USA; 9 Alpert Medical School, Brown University, Providence, USA

**Keywords:** HIV, Bioethics, Biobanking, Research, Policy, Young people, Caregivers

## Abstract

**Introduction:**

Biobanking is common in research involving young people living with HIV (YPLHIV). The ethics and policies guiding this practice require careful consideration, especially given the population’s multiple vulnerabilities, like age, HIV status, and limited resources. We examined how well the perspectives of YPLHIV and other stakeholders are reflected in current policies in sub-Saharan Africa, aiming to inform more ethically grounded policy development for specimen biobanking.

**Methods:**

We conducted a review of biobanking-related policy documents from sub-Saharan Africa, primarily from the East African region, and compared them to qualitative findings from interviews with YPLHIV in western Kenya, their caregivers, and subject matter experts (SMEs) regarding perspectives on biobanking. We synthesized the policy and interview data to identify key similarities, differences, and gaps. Themes were organized into three main categories related to biospecimens: (1) collection and analysis, (2) storage and identification, and (3) testing and sharing.

**Results:**

Interviews were conducted with 99 participants – 40 YPLHIV, 20 caregivers, and 39 SMEs (community leaders, healthcare providers, clinical researchers, social scientists, international research experts, and laboratory experts). Participants across all groups stressed the importance of informed consent, results dissemination, confidentiality, transparency, and secure storage. Additional themes included concerns about long-term storage, unauthorized use, sharing with ill-intentioned individuals, requests for participant benefits, expressions of trust in researchers, and disagreement over using identifiers in biospecimen labeling. Interview themes were reflected to varying degrees in policy documents. Our policy search revealed articles from 12 countries, published between 2004 and 2023. All countries addressed consent and confidentiality (*n* = 12) and most covered results dissemination (*n* = 11) and biospecimen sharing (*n* = 9); fewer addressed participant benefits (*n* = 4), labeling (*n* = 4), and direct guidance on use, location, and duration of storage (*n* = 4). Some gaps between stakeholder views and existing policies were evident.

**Conclusion:**

Perceptions of research involving biobanking among African YPLHIV were mixed, revealing inconsistencies in participants’ responses, and highlighting gaps between these perceptions and existing policies, which were often limited, outdated, and incomprehensive. Findings highlight the need for clear, timely and inclusive policy updates that reflect stakeholder input, particularly as research involving this vulnerable population continues.

**Supplementary Information:**

The online version contains supplementary material available at 10.1186/s12910-026-01421-7.

## Introduction

Young people represent a growing proportion of people living with HIV worldwide and a uniquely vulnerable population. In 2022 alone, almost 500,000 young people, aged 10–24 years, newly acquired HIV [1]. Compared to adults, young people living with HIV (YPLHIV) tend to have poorer HIV-related outcomes, including lower rates of viral suppression and reduced likelihood of long-term immunologic recovery [2, 3]. YPLHIV also experience high rates of HIV drug resistance [4, 5], which can increase their risk of HIV-related mortality [6]. In addition, YPLHIV experience unique vulnerabilities with regards to social determinants of health, including bullying and discrimination at schools, stigmatization at home, violence and prosecution with regards to sexual and gender orientation, and exacerbations of poverty and malnutrition [7–9].

Biobanking – the processing, storing, and distributing of biospecimens and associated data for research and clinical care [10] – can be invaluable for improving medical care, including that related to HIV. However, it raises ethical questions related to consent, confidentiality, ownership, governance, return of results, benefit sharing, and specimen storage and identification [11]. These issues are especially important when biobanking specimens from young people, a vulnerable population requiring special protections, and in African settings, where imbalanced international research partnerships may threaten recapitulation of colonial-era dynamics [11]. Moreover, widespread biobanking of specimens is a relatively new phenomenon in many of these settings [12].

Incorporating the perspectives of research participants and other stakeholders – including caregivers, community leaders, healthcare providers, clinical researchers, social scientists, international research experts, and laboratory experts – in the research process is critical to ensuring biobanking is conducted ethically. Few studies have examined ethical issues related to research with biospecimens in African countries [13–19], and even fewer have addressed these issues specifically in relation to YPLHIV [18, 19]. Furthermore, national ethics guidelines on research involving biospecimens and biobanking vary widely across African countries and offer limited consensus on key issues, such as consent, collection, analysis, storage, identification, and testing and sharing of biospecimens, especially in relation to the unique perspectives, vulnerabilities, and needs of YPLHIV [20–33].

In this study, we reviewed available policies from African countries that provide guidance on biobanking research involving young people and compared them to perspectives on specimen biobanking elicited through qualitative interviews with Kenyan YPLHIV, their caregivers, and subject matter experts (SMEs). This synthesis aimed to assess the extent to which stakeholder concerns were reflected in existing regional policies so that these policies can be improved to better address stakeholder priorities and inform ongoing research efforts in Kenya.

## Methods

### Study setting and design

This study was conducted in western Kenya within the Academic Model Providing Access to Healthcare (AMPATH), a partnership between Moi Teaching and Referral Hospital (MTRH), Moi University School of Medicine, and a global consortium of academic health centers led by Indiana University. AMPATH provides comprehensive care to over 110,000 people living with HIV, including nearly 10,000 YPLHIV, across more than 300 clinical sites in Kenya [34, 35]. We reviewed published policies addressing ethical considerations related to biobanking and conducted semi-structured interviews with participants to obtain their perspectives on this topic. We subsequently compared and synthesized findings to determine if participant priorities and concerns related to research involving biospecimens were adequately reflected in regional guidelines.

### Policy review

We conducted a review of policies and guidelines regarding ethical considerations involved in the collection, analysis, storage, identification, testing, and sharing of biospecimens for research purposes. These policies came from countries in sub-Saharan Africa, primarily from the East African region. Scholarly databases were mined using the following search terms with Boolean operators: *bioethics*, *research regulation*, *biospecimen*, *data sharing*,* biospecimen storage*,* future use*,* Sub-Saharan Africa*,* East Africa*,* Kenya.* Guidelines published by the Kenya National Commission for Science, Technology and Innovation (NACOSTI) and AMPATH Consortium institutions were also reviewed. Three authors (EG, DL, NC) conducted the search and exported policy documents to an Excel spreadsheet. AC and MS evaluated each policy for clarity, operational relevance, and regulatory alignment. SS then analyzed articles and extracted key themes, which were reviewed by AC and KG. The policy search was completed in November 2023. SS completed a comprehensive review of the policy documents and KG, SS, and AC subsequently completed the synthesis of policy and interview findings. Identified policies were downloaded and housed in a password-protected shared drive on the secure campus of the Icahn School of Medicine at Mount Sinai. Data extraction and analysis records were housed with the identified policies.

### Semi-structured interviews: participant recruitment

The qualitative interview methodology has been described in depth elsewhere [18, 19, 36]. Participants included four different groups, all residing in Kenya:

1. *YPLHIV* *enrolled in a parent study*. YPLHIV were initially enrolled in 2010 for an ongoing parent study assessing antiretroviral therapy (ART) adherence and HIV drug resistance in a cohort to optimize treatment monitoring and outcomes [6, 37, 38]. The parent study’s eligibility criteria included: (1) perinatally-acquired HIV infection, (2) 14 years or younger at enrollment, (3) on or starting first-line non-nucleoside reverse transcriptase inhibitor-based ART regimens, and (4) receiving HIV care at one of four AMPATH clinics. For the current study, random sampling was used to recruit YPLHIV from the parent study, and participants were eligible if they were: (1) enrolled in the ongoing parent study, (2) 10–24 years of age, and (3) aware of their HIV status. Participants were either contacted by phone or approached at the clinic during routine medical visits. All participants who were contacted agreed to partake in the study.
2. *Caregivers of YPLHIV enrolled in the parent study* were enrolled to share perspectives tied to their experience providing consent and other supports for their children involved in research. Caregivers of YPLHIV not enrolled in the parent study were not included given they did not have this experience to draw from*.* Caregivers were recruited via random sampling and were eligible to participate if they were: (1) 18 years of age or older, (2) a caregiver of a YPLHIV enrolled in the parent and current study, (3) aware of their child’s HIV status, and (4) apprised of the care and research participation of the YPLHIV. Caregivers were contacted by phone or during the young peoples’ routine clinical visit.
3. *YPLHIV with no history of research participation* were enrolled to provide an alternative perspective to research-experienced YPLHIV, whose responses could be biased by their experience and existing relationships with current and prior study teams. These participants were randomly sampled from clinical appointments at the MTRH Rafiki Center for Excellence in Adolescent Health, a clinic providing comprehensive HIV and other health services to adolescents and young adults. Young people were eligible if they were: (1) 10–24 years of age, (2) living with HIV and aware of their status, (3) receiving care at the Rafiki Center, and (4) not involved in current or prior clinical research studies per self-report. Caregivers were also required to know the HIV status of their child participating prior to enrollment. This group of YPLHIV was combined with the first group for the purposes of this analysis.
4. *SMEs* were enrolled as additional stakeholders with experience working with young people and/or involved in biobanking-related research. SMEs were identified by the research team and were eligible to participate if they were (1) 18 years of age or older, and (2) a member of one of the following groups: community leaders (village elders and chiefs), AMPATH community advisory board members, healthcare providers working with young people, local or national Institutional Review Board (IRB) members, researchers from the AMPATH Research Network [39], research laboratory leaders (assisting in protocol development for the processing, storage, and use of biospecimens in research; also, often conducting phlebotomy directly with participants), or Kenyan government or policy representatives.

Those who did not provide written informed consent, or, in the case of those younger than 18 years old, those who did not provide assent with consent from a caregiver, were excluded from the study. Participants were enrolled until thematic saturation was achieved within each participant group.

### Data collection and analysis

All participants underwent semi-structured interviews, exploring their concerns about and recommendations for biospecimen collection, analysis, storage, identification, testing, and sharing. Interview guides were informed by a literature review and the study team’s long-standing experience working with YPLHIV in Kenya. Interview guides have been previously published elsewhere [36]. Interviews were conducted by two experienced Kenyan facilitators (one male, one female) in either Kiswahili or English, depending on participant preference. Interviews were conducted in private rooms and no observers were present. Interviews were audio-recorded, transcribed, and translated into English if conducted in Kiswahili. Study participants were assigned a unique study ID. Study participants were not asked to identify themselves during the interviews. Audio recordings and transcripts were kept by the study team on password-protected computers and files that remained in a locked study office on the secure campus of the Moi Teaching Referral Hospital with access to study data only allowed by study investigators and other study personnel.

Data from both groups of young people, those with past research participation and those without, were analyzed together. All SME categories were also analyzed together. Two researchers (HK and EG) led the thematic analysis based on a coding framework derived deductively from interview questions and inductively from interview responses. The researchers independently extracted data using the qualitative software program NVivo, version 12 [40]. Their work was reviewed, and conflicts were resolved by JA and AC. Interview responses were grouped into three categories: (1) collection and analysis of biospecimens, (2) storage and identification of biospecimens, and (3) testing and sharing of biospecimens. Within each category, interview quotes were further grouped into themes and sub-themes.

### Ethical approvals

This study was approved by the Lifespan IRB in Providence, RI, USA (Package number: 1452141), Icahn School of Medicine at Mount Sinai IRB in New York, NY, USA (study number: 20-01177), and the Moi University/Moi Teaching and Referral Hospital’s Institutional Research and Ethics Committee (IREC) in Eldoret, Kenya (approval number: 0003689). Additional approval was received from the National Commission for Science, Technology and Innovation (NACOSTI), a Kenyan government research regulatory body (reference number: NACOSTI/HW/3/1/16). All participants aged 18 years and older provided written informed consent to participate in study interviews, and participants under the age of 18 provided written assent accompanied by caregiver consent. Detailed, standardized informed consent and assent forms, written in either Kiswahili or English, were read aloud prior to interviews with adequate time for questions and clarifications. Written consent and assent, when required, were obtained by trained, bilingual research assistants with experience recruiting YPLHIV in clinical research studies.

## Results

### Policy overview

A total of 14 policy documents from 12 countries, published between 2004 and 2023, met eligibility criteria [20–33]. Botswana, Ethiopia, Nigeria, Rwanda, South Africa, Sudan, Tanzania, Uganda, Zambia and Zimbabwe offered one document each, and Kenya and Malawi offered two. Document types varied; most were ministry of health and ethics committee guidelines (10), while some were standard operating procedures (2). There was also one act and one policy statement. The policies addressed a range of topics aligned with the three categories into which interview findings were ultimately grouped. Within the biospecimen collection and analysis category, policies discussed informed consent, results dissemination, and the delineation of participant benefits. Within the other two categories, they addressed storage and future use, confidentiality, biospecimen labeling, and sharing biospecimens with others. Areas of overlap between major interview themes and policies are outlined in *Supplemental Table 1*.

### Semi-structured interviews with YPLHIV, caregivers, and SMEs

Semi-structured interviews were conducted with 99 participants (52% female), including 40 YPLHIV (median age 18, age range 11–24 years, 50% female), 20 caregivers (70% female), and 39 SMEs (44% female, 18 community leaders, 10 healthcare providers, 6 clinical researchers, 3 social sciences researchers, 1 international research expert, and 1 laboratory expert).

What follows is a synthesis of findings from the semi-structured interviews according to the three outlined categories, and the overlap, or lack thereof, with regional policy documentation. 

## Collection and analysis of biospecimens (Table 1)

### Concerns regarding the collection and analysis of biospecimens

Members of all participant groups shared concerns about biospecimen collection. To avoid excessive blood draws, all participant groups recommended improved coordination between clinical and research teams; SMEs specifically recommended phlebotomist re-education, and YPLHIV recommended that researchers use existing biospecimens rather than collect additional samples. Some YPL HIV and caregivers were less concerned about collection, noting that collection does not cause undue harm to participants, especially if they coincide with routine blood draws and are spaced out appropriately. Some of these concerns were considered in policy documents, while others were not mentioned. Of note, none of the reviewed policy documents addressed participants’ concern about excessive blood draws.

**Table 1:** Collection and analysis of biospecimens

Theme	Sub-Theme	Illustrative Quote	Policy Intersection
Concerns	Frequent biospecimen collection	“I don’t think it’s very nice if somebody’s sample is taken today for their routine clinic and then in 2 week’s time or the following day the research team calls this patient to draw the same sample.” – Female, SME, healthcare provider	-None
		“There was a research [project] that took [my child’s] blood samples twice in a single day…and when I came back they did not even tell me why they took his blood twice.” – Female, caregiver	
	Biospecimens may be used for unauthorized purposes	“My worry is that you collect biospecimens and you put them out there and anybody with any intention shows up and they want to use it for things that are totally unrelated to what the initial participant was involved in.” – Male, SME, IRB	Ethiopia, 2014 [21]; Kenya, 2020 [23]; Malawi, 2012 [25]; Rwanda, 2009 [27]; South Africa, 2017 [28]; Tanzania, 2023 [30]; Zambia, 2013 [32]
		“Some people believe that their blood could be taken somewhere and that person bewitches them and messes up their lives. There are some who believe that their blood would be taken [for] satanic things.” – Male, YPLHIV, < 18 years, research-experienced	
Conditions for providing biospecimens	Informed consent must be given	“I don’t have any concern with research that analyzes blood or tissues or anything, so long as the participant is well informed. You see the consent must be gotten after full disclosure of what is going to happen. And it must be voluntary, and the person must be willing to participate.” – Male, SME, clinical researcher	Botswana, 2005 [20]; Ethiopia, 2014 [21]; Kenya, 2017, 2020 [22] [23]; Malawi, 2007 [24]; Rwanda, 2009 [27]; South Africa, 2017 [28]; Sudan, 2008 [29]; Tanzania, 2023 [30]; Uganda, 2014 [31]; Zambia, 2013 [32]; Zimbabwe, 2004 [33]
		“Giving out your blood is an option, it is not a must. I think if you have an agreement, that’s how you can give out your blood.” – Female, YPLHIV, > 18 years, no past research	
	Must yield benefits to participants	“If you collect blood, after screening…if it contains, let’s say, impurities or diseases, those diseases may have not been detected by the participant, but that research would have sped up the diagnosis and led to early treatment.” – Male, YPLHIV, > 18 years, no past research	-None
		“I don’t have fears because I asked them about it, and they told me they’re going to test if the drugs are utilized well, and if is not utilized well, they can know how to help early. They were going to test the blood and virus levels. If it’s not going well, they can change the drugs.” – Female, caregiver	
	HIV-specific results must be shared	“If you find something that is important, a new finding from their sample, then it’s good to actually give feedback to let them know. For example, we tested your blood and we found some problems; we found that the disease has not progressed or you are developing some form of resistance, so this feedback should be given to both the patients and [their] healthcare providers.” – Male, SME, clinical research	-None
		“If blood is drawn from my child, they should tell her because maybe her viral load is high because she is not taking the medication as she should…If everything is okay from the tests, it motivates her to properly take the medication.” – Female, caregiver	
	Findings must be transparent	“For any project or research, whoever was involved should get that feedback. [If] a blood sample was taken and they never got that report, they might be left wondering [if] their blood was taken for a devious purpose. It is for the benefit of the researcher, the institution and whoever the blood sample was taken from.” – Male, SME, chief	Botswana, 2005 [20]; Ethiopia, 2014 [21]; Malawi, 2012 [25]; Rwanda, 2009 [27]; Uganda, 2014 [31]; Zimbabwe, 2004 [33]
		“They are supposed to share the information so as to properly understand the research.” – Male, YPLHIV, > 18 years, research-experienced	

### Conditions for providing biospecimens

Members of all participant groups agreed that informed consent was required for biospecimen collection; this was also well-represented in policies from each country included in the review (*n* = 12) [20–33]. Policies required that informed consent be completed prior to biospecimen collection [24, 28, 32], and that consent documentation contain information on storage, future use, and sharing of biospecimens [20–33]. Following biospecimen collection and analysis, members of all participant groups emphasized the importance of sharing results with participants, especially those related to HIV viral load and drug resistance; most countries included in the review (*n* = 11) had policies for sharing research findings with study participants [20–25, 27–33]. Kenyan and Ethiopian policies indicated that results sharing should be general, rather than individual [21, 23]. Rwandan policy stated that participants must be notified if research findings would be widely disseminated in publications and conferences [27]. Zambian policy specified that results must first be discussed locally before being distributed internationally [32].

In the case of abnormal findings resulting from biospecimen testing, SMEs and YPLHIV emphasized facilitating appropriate linkage to care. Combining results dissemination with ‘guidance’ and ‘counseling’ was thought to be highly ethical, a valuable opportunity for follow-up, a means of building rapport with participants, and beneficial to future recruitment efforts. Some participants outlined conditions for results dissemination; SMEs felt that sharing ‘incurable results’ would just cause ‘unnecessary stress.’ YPLHIV thought that sharing HIV-related results would risk unintended disclosure. Fewer countries in this analysis (n = 4) had policies requiring researchers to outline expected benefits from biospecimen collection and analysis. Tanzanian, Kenyan, and Ethiopian policies required that expected benefits be clearly articulated in consent forms [21, 22, 30]. In Kenya and Ethiopia, polices required this discussion to also include expected benefits to the community as a whole [21, 22]. South African policy stated that a study’s community benefits must be included in the protocol [28]. 

## Storage and identification of biospecimens (Table 2)

### Concerns among participants

Many participants shared concerns about biospecimen storage. Most policies reviewed (*n* = 9) required storage procedures to be discussed in consent forms [20, 21, 23, 25–27, 29, 31, 33]. All three participant groups felt that biospecimens should be disposed of after use and that future analyses should collect new biospecimens; this practice was viewed as protective against misuse. Policies from Sudan and Zimbabwe gave participants the right to have their biospecimens destroyed [29, 33]. Both SMEs and YPLHIV requested more information about storage location, expressing concerns about international storage. SMEs and caregivers questioned whether storage provided any benefit to participants, citing concerns that storage may delay benefits and results reporting to YPLHIV. SMEs also shared concerns about proper labeling and disposal of biospecimens and adherence to ethics principles. 

**Table 2:** Storage and identification of biospecimens

Theme	Sub-Theme	Illustrative Quote	Policy Intersection
Concerns	Biospecimens should not be stored	“Why should they keep that blood for [a] long time? For example, if a researcher has to label some blood, then after 5–6 years that researcher is maybe fired or has retired, then the other researcher just picks that blood [and] maybe he/she doesn’t know the consent of that blood. It may mislead people…so I’d recommend that if they want to use specific blood, they use that blood sample and when they are finished, they destroy it. Then [when] they come up with more research, they just collect more blood samples.” – Male, SME, community advisory board	-Malawi, 2012 [25] -Nigeria, 2013 [26] -Zambia, 2013 [32]
		“I have concerns because why and where are they keeping it? They will call me to tell me that the child’s blood is like this and this, [so] what is the point of storing it yet they have already used it?” – Female, caregiver	
Conditions for storage of biospecimens	Informed consent must be given	“So long as you are following research principles, they have consented and they have disclosed fully what they are going to do with that blood, [there is] no issue whatsoever.” – Male, SME, healthcare provider	Botswana, 2005 [20]; Kenya, 2020 [23]; Sudan, 2008 [29]; Tanzania, 2023 [30]; Zambia, 2013 [32]; Malawi, 2012 [25]; Zimbabwe, 2004 [33]
		“It is best that the research party be upfront with what they want to do with the blood, then plans [for] the future, anything pertaining using the sample should be aired out to the participant.” – Male, YPLHIV, > 18 years, no past research	
	Storage must be safe and secure	“It depends on the laboratory [where] it has been stored and [the] security of the samples. Because the world is changing and someone can take that blood and inject it in others to be used as a weapon in warfare. I don’t think the blood of the infected should be stored for long in the laboratories. If they are done with the blood, they should dispose [of] it because the blood samples are contaminated and are dangerous already. Someone might want revenge on someone and use that blood as [a] weapon.” – Male, SME, MTAA (neighborhood) community leader	Botswana, 2005 [20]; Kenya, 2020 [23]; Malawi, 2012 [25] -Nigeria, 2013 [26]; South Africa, 2017 [28]; Tanzania, 2023 [30]; Uganda, 2014 [31]; Zambia, 2013 [32]; Zimbabwe, 2004 [33]
		“[The researcher] should assure the subject that the samples will be kept safely and stored correctly…They should just confirm privacy [and] storage.” – Male, YPLHIV, > 18 years, no past research	
	Storage must benefit research	“I do not have any worries because I believe in the research and I know that [it] will bring us closer to solving this problem. I believe with time we might discover the medicine.” – Male, SME, chief	Kenya, 2020 [23]; South Africa, 2017 [28]; Tanzania, 2023 [30]; Uganda, 2014 [31]; Zimbabwe, 2004 [33]
		“I do not have concerns, so if they have already taken the sample and they want to do research to find out some things, there is no problem.” – Female, caregiver	
Name- based labeling of biospecimens	Offers easy identification	“When they use the name of the participant, it will be easy to identify whose blood sample it is.” – Male, YPLHIV, < 18 years, no past research	-None
		“The blood should be under the child’s name because if you use a code it [could] confuse but for a name you’ll definitely get it.” – Female, caregiver	
	Offers easy follow-up	“Name usage is better for follow up; my contacts should be there in case they want to do future research. They can easily trace me.” – Male, SME, MTAA community leader	-None
		“When you find out the outcome of the research, it might be easier to contact the client, to inform them of the research study.” – Male, YPLHIV, > 18 years, no past research	
	Risks inadvertent disclosure	“Keeping the name of that person might leak the information about a client. For example, if someone comes here and sees the blood sample and sees the name John, he goes out and tells John his blood [is] in the blood bank and asks when he removed it and that he must have a problem. That is not good. It is good to keep secrets.” – Male, SME, chief	-None
		“Obviously when you indicate the name in the specimen, people will know your status and wherever you go people will discriminate you or just start discussing…you.” – Female, YPLHIV, > 18 years, no past research	
Number – based labeling of biospecimens	Offers confidentiality	“The best way would be to store it without the personal identifiers so that you protect the participant, in terms of their privacy, in terms of confidentiality.” – Female, SME, international research expert	-None
		“If they store with the numbers they maintain privacy.” – Female, YPLHIV, > 18 years, research-experienced	
	Risks difficult identification	“If they use the number it will be hard to identify.” – Male, YPLHIV, < 18 years, research-experienced	-None
		“If you give random numbers, how will you know the results of that person? So, you may bring results which are not mine or the feedback may be given to a different person.” – Female, caregiver	
	Risks specimen confusion	“The numbers might get mixed up. So, the doctor might come and take blood for another person. Maybe the person might get stigmatized. So, it might lead to that social effect, depression and stuff because of the wrong results.” – Female, YPLHIV, < 18 years, no past research	-None
		“For a number, you can write it and maybe you miss a digit.” – Female, caregiver	

### Support and conditions for biospecimen storage

Participants in all groups offered support for biospecimen storage, believing that storage could reduce the need for future blood draws and that additional research on stored specimens could help others. Some participants across all groups expressed support for storage, provided that participants gave informed consent for storage specifically, confidentiality was maintained, and/or the storage would benefit research. Policies from all countries included in the review recommended the use of anonymized biospecimen storage to ensure confidentiality [20, 21, 23–33], and several policies required confidentiality procedures to be outlined in the research protocol, including who would have access to identifying information [20, 24, 30]. In Tanzania and Uganda, policies required that consent forms be the only documents linked to individual participants [30, 31].

### Labeling of biospecimens: names or numbers

All participant groups discussed two methods of identifying stored biospecimens: name-based and number-based labeling; yet, only four countries in this analysis had policies regarding labeling biospecimens [21, 25, 26, 28]. Participants in all groups said name-based labeling could allow for easy identification and follow-up, and would prevent the loss of samples; however, they also worried that it risked inadvertent disclosure of HIV status and other sensitive information. SMEs specifically viewed name-based labeling as unethical and unprofessional, and believed it risked fostering corruption and distrust in researchers. YPLHIV were concerned that the risk of disclosure from identified biospecimens might dissuade young people from participating in future studies. An additional concern, held by YPLHIV and caregivers only, was that participants may share the same name causing possible confusion of biospecimens.

Many participants across all groups thought that number-based labeling was the best way to preserve participants’ privacy and confidentiality; this was also supported by several policies reviewed. South African policy required clear, accurate labeling for transport and storage [28]; and policies from Ethiopia, Malawi, and Nigeria required anonymized biospecimen labeling [21, 25, 26]. SMEs thought that number-labeling would allow for unbiased analyses, and confidential publishing and sharing of data with collaborators. Interestingly, some participants from all groups worried that number-based labeling may make biospecimen identification more difficult, could lead to a loss of biospecimens, and would present disclosure risks as members of the study team would still know which number corresponded with which participant. YPLHIV were concerned that these risks may require repeat blood draws.

## Testing and sharing of biospecimens (Table 3)

### Concerns, justifications, and conditions for additional testing of biospecimens

Participants across all groups expressed concerns about future testing of biospecimens. Future use was well-represented in reviewed policies, with a majority requiring consent forms to discuss future use of biospecimens in detail [20–23, 25–31, 33]. Participants in all three groups shared concerns about the purpose of additional testing and thought that an additional informed consent process should occur. Policies from Ethiopia, Kenya, Rwanda, and Uganda supported this sentiment, requiring additional consent for further testing [21, 23, 27, 31]. SMEs and YPLHIV emphasized that participants still own their biospecimens and stressed the need for continued confidentiality. SMEs thought that the IRB would first need to approve any additional testing. Other participants, across all groups, viewed the original consent as sufficient approval for future testing, with some SMEs arguing that ownership of biospecimens was transferred to the researcher after collection. There was a worry among both SMEs and YPLHIV that contacting participants to request additional testing approval might cause unnecessary distress. Several policies supported these perspectives; Nigerian and South African policies allowed for broad consent to cover secondary uses of biospecimens [26, 28], and Tanzanian and Ugandan policies, following consent for future use, allowed institutions to make decisions regarding use on behalf of participants [30, 31]. Participants across all groups shared conditions for future testing, including informed consent before testing, notifying participants of future results, and re-consenting adults who provided initial consent when they were minors. These efforts were viewed as a sign of goodwill, building strong relationships between researchers and participants, and preventing feelings of being ‘cheated’ or ‘taken advantage of’ among participants. Policies in Sudan and Zimbabwe supported these perspectives, indicating that participants have the right to decide how their biospecimens are used in the future [29, 33].

**Table 3:** Testing and sharing of biospecimens

Themes	Sub-Theme	Illustrative Quotes	Policy Intersection
Additional Testing Concerns	Concern for unauthorized testing	“I would be concerned about reuse of my blood. Are you likely to use my blood for something other than what you first collected it for? And if you are going to use it for something else, what is that something else? Do I have control over what you can use it for? And once you use it and there are benefits coming from it, am I going to benefit?” – Female, SME, international research expert	-Ethiopia, 2014 [21]
		“It is not good because that blood sample was specifically for research; it wasn’t [meant] to be used for any other purpose.” – Female, caregiver	
Additional Testing Justifications	Additional tests will aid research	“If it is going to help in research, it should be used.” – Male, SME, MTAA community leader	-Ethiopia, 2014 [21]
		“You give out your blood so that they can do research on it. Even if they do things other than the original reasons, that is still research. And it is okay.” – Female, YPLHIV, < 18 years, research-experienced	
	Participants already gave informed consent	“Once that person has volunteered to give you blood, it is not limited to the objective that you have at that moment, it might be broader research or something unplanned. Once you are within your context I don’t see why you need to go back to that person.” – Male, SME, chief	-Ethiopia, 2014 [21]
		“I feel because the participant has agreed to the terms and conditions of giving out a sample, any way it is used…should not be of any concern to him.” – Male, YPLHIV, > 18 years, no past research	
	Researchers already in possession of biospecimens	“Once the public has donated blood for testing, it is the end of that person to own the blood, the doctor should not go back and seek consent to check for other diseases. There is no need because the blood is already with the doctor and now it is owned by the hospital to do anything they wish.” – Male, SME, MTAA community leader	-Uganda, 2014 [31]
		“I felt it’s better if it’s stored for the future because if we say it shouldn’t be used, how will it be helpful? It has already been drawn and you can’t put it back in the body, let it just be used for whatever it can be used for.” – Male, caregiver	
Conditions for additional tests on stored biospecimens	Participants should be contacted prior to testing given possible medical implications	“Maybe you were testing for a disease and it was found in my blood and you did that research without my [knowledge]; you will call me and tell me we did a different test and we found this. What do you think I will feel? Of course I will feel bad. I think you should notify me first and tell me we want to use your blood sample to do different research.” – Male, YPLHIV, > 18 years, research-experienced	Botswana, 2005 [20]; Kenya, 2020 [23]; Zimbabwe, 2004 [33]
		“How I am doing health wise, they should inform us.” – Male, caregiver	
	Participants should be contacted prior to testing to provide informed consent	“One, there needs to be a deliberate effort to ensure that they can reach the adolescents and ask whether they still hold the same views as when they gave their permission of storage. If they have changed then they should be given an opportunity to not participate. Two, since it is the parent who gave the initial consent, when they get to 18 years and can give their own consent, there should be room for them to be re-consented.” – Male, SME, IRB	Botswana, 2005 [20]; Kenya, 2020 [23]; Malawi, 2012 [25]; Tanzania, 2023 [30]; Zimbabwe, 2004 [33]
		“I gave out my blood for the viral load. I [think] that my blood was disposed of. But now when they come and tell me that they want to do blood resistance, they should consent me so that I know that they are doing this and it is for this reason.” – Female, YPLHIV, < 18 years, no past research	
Concerns related to the external sharing of samples	Concerns about how recipients will use shared biospecimens	“You should be concerned because you want to know what kind of research they are doing, and if it benefits society or you as a person.” – Female, YPLHIV, > 18 years, no past research	-None
		“I don’t know what those others are going to do with it unlike here I know what it is for. That is my worry.” – Female, caregiver	
	Concerns that biospecimens could infect or contaminate	“Not all organizations have a good opinion about the blood samples; some would use that blood sample in a bad way such as creating a virus…especially if it’s an adolescent because that blood is very contaminated and could be a bioweapon.” – Male, SME, community advisory board	-None
		“They can take blood that is infected and use it or transfuse someone who is negative and at the end will be infected.” – Male, YPLHIV, < 18 years, no past research	
Justifications for external sharing of samples	Participants had already provided informed consent	“If at first the participant gave you permission to sample the blood and run tests on it say for HIV and also gave you permission to store it. I think if you want to use it for other types of research then that is okay.” – Male, SME, chief	Kenya, 2020 [23]; Ethiopia, 2014 [21]; Zimbabwe, 2004 [33]
		“I don’t think they should contact me; I have already donated and I have signed that this blood will be used for research. That research may be country-wide and it will be for the benefit of all of us.” – Female, caregiver	
	Researchers already in possession of biospecimens	“When I come to the hospital, it is not limited to carry out one test, they can have a broad objective, and they don’t need consent from you to do that. The only time they will need consent is when they want another sample from my body.” – Male, SME, chief	Ethiopia, 2014 [21]
		“The researchers have the permission now because I have already given my blood and left. You now have the authority, not me.” – Female, caregiver	
	Trust in researchers and their research	“It should be upon the researchers because the participant only knows so much about the research, so it won’t be a strategic move to involve them in a certain development or expansion of the research.” – Male, YPLHIV, > 18 years, no past research	-None
		“Our association ends here but when you associate with other researchers, it will be between you and them so I don’t think that we can interfere. If he feels that it will be in the best interest of the participant, he should share, but if he feels that it won’t help the participant then he should defend the participant by not sharing because he is the one who knows if sharing will benefit the participant.” – Male, caregiver	
Conditions for external sharing of samples	Participants should be informed about the purpose for sharing biospecimens	“If along the way you decide you want to give it to somebody else to use it for a different purpose, or even for the same purpose, I think it could be essential for me to know and re-consent so that [I] am able to monitor what is happening to my blood.” – Female, SME, international research expert	Botswana, 2005 [20]; Kenya, 2020 [23]; Zimbabwe, 2004 [33]
		“It is important to know why you are giving a blood sample and what type of research is being conducted and the purpose behind the research.” – Male, Caregiver	
	Confidentiality should be maintained	“I don’t think there will be much [concern with]…sharing with other researchers and maybe to other governmental institutions or non-governmental institutions as long as they keep their confidentiality.” – Female, SME, healthcare provider	Kenya, 2020 [23]; South Africa, 2017 [28]; Zimbabwe, 2004 [33]; Botswana, 2005 [20]
		“If they are confidential [and] keep it anonymous then I do not have an issue with it being shared.” – Female, YPLHIV, > 18 years, no past research	

### Concerns, justifications, and conditions for sharing biospecimens with other researchers or organizations

Participants in all groups expressed concerns about sharing biospecimens, worrying that they could be used to harm others, such as in the creation of a ‘virus’ or ‘bioweapon.’ They also had hesitations about sharing biospecimens with the government or other researchers, hospitals, or organizations outside of those that initiated the research. YPLHIV specifically preferred that their biospecimens be kept locally. Nine of the countries reviewed (Botswana, Ethiopia, Kenya, Malawi, South Africa, Sudan, Tanzania, Uganda, and Zambia) had policies related to sharing biospecimens [20–24, 28–32], with South Africa and Uganda also requiring a material transfer agreement (MTA) to be established between institutions prior to sharing.

Other participants in all groups thought the original consent gave researchers a ‘broad objective’ to do what they wanted with the biospecimens in their possession and effectively transferred ‘authority’ to them. These participants trusted researchers to act in their best interest and cautioned that excessive consultation of participants would hinder research progress. In a similar vein, reviewed policies from Tanzania and Uganda gave institutions the right to transfer biospecimens on behalf of participants [30, 31].

Participants in all groups also offered recommendations for sharing biospecimens, including informing participants of the purpose for sharing, maintaining confidentiality, and ensuring results are shared with participants. SMEs thought sharing was permissible, as long as the research protocol was followed and participants were contacted prior to sharing; these efforts were viewed as important for maintaining the researcher-participant relationship and not hindering future research recruitment. Caregivers thought sharing biospecimens was acceptable so long as a clinic authorized the sharing, the biospecimens were stored properly, and testing was conducted in a clinic setting. Policies from Botswana, Kenya, and Malawi specified that consent forms include information about sharing biospecimens [20, 22–24], and Ethiopian and Sudanese policies stated that participants have the right to decide if their biospecimens are shared externally [21, 29].

## Discussion

There is a paucity of studies examining ethical issues in biospecimen research in African countries [13–19], particularly those focused on YPLHIV [18, 19]. To address this gap, we explored the perspectives of young people and other stakeholders and assessed how well these views are reflected in policies from 12 African countries. Our findings aim to inform policy improvements that better align with stakeholder priorities and promote ethically grounded research involving biospecimens from this vulnerable population. Participants’ insights – at times divergent, and policies – often limited or inconsistent – revealed several gaps between stakeholder perspectives and existing guidance. These findings highlight the need to prioritize key ethical concerns while also addressing broader stakeholder input. Our multi-method qualitative approach supports more responsive, stakeholder-informed policy development.

Input from participants was rich and, in many cases, aligned across groups, though notable differences did emerge. Themes within three defined categories – (1) collection and analysis, (2) storage and identification, and (3) testing and sharing – revealed that informed consent, results dissemination, confidentiality, and secure storage were important priorities for participants in all groups. Notable sub-themes included concerns about long-term biospecimen storage, unauthorized use, and sharing of biospecimens with ill-intentioned individuals. While some requested assurances of participant benefits, others expressed faith that researchers would act in participants’ best interest. There was also disagreement over the use of identifiers in biospecimen labeling, with participants primarily weighing the risk of unintended disclosure against the advantages of easy identification and follow-up.

Consensus on many issues among the interviewed groups can guide policy development. However, the observed inconsistencies highlight the importance of considering whether, and to what extent, all perspectives should be incorporated. They also point to the need for larger studies to better characterize which subgroups hold which views. Such data could inform broader guidelines that include more targeted considerations for specific participant populations whose biospecimens are being collected, as well as their support systems. These efforts place these populations as *collaborators* in the biobanking research process, as opposed to just *participants*, a perspective change advocated for in a prior study [41].

These themes were reflected to varying degrees in the policy document review, which revealed several notable findings and highlighted key gaps. First, most available policies were outdated and had not been recently revised, highlighting the need for updates that reflect current ethical considerations and research practices, a concern also noted in a prior study [42]. Second, the comprehensiveness of policies varied across countries. All reviewed countries (*n* = 12) had policies addressing confidentiality and informed consent. Most included statements on results dissemination (*n* = 11) and sharing biospecimens with other researchers or institutions (*n* = 9). However, fewer policies addressed delineation of participant benefits in study documents (*n* = 4) or providing guidance on labeling biospecimens (*n* = 4). Policies addressing biospecimen storage and future use were typically framed within the context of informed consent, with few offering specific guidance on *how* biospecimens should be used, or *where*,* how*,* and for how long* they should be stored. Third, policies often diverged from one another in scope and emphasis, reflecting a lack of regional consensus. Fourth, and most critically, several policies diverged from the perspectives shared by participants in our interviews, pointing to disconnects between policy and stakeholder concerns that warrant further attention.

More policies must be developed and updated to address identified gaps, with greater integration of stakeholders’ perspectives. For example, concerns about improper storage, misuse, and inequitable benefit distribution were frequently raised by Kenyan YPLHIV, SMEs, and caregivers in our interviews, yet these issues were minimally represented in existing policies. Similar concerns have been observed elsewhere in the region. A prior qualitative study in South Africa reported that adult participants wanted more transparency about “where and why” their blood was being stored, expressed worries about biospecimen export and misuse, including references to “satanic rituals,” and requested a “portion of the profit” from resulting research, echoing concerns in our study [14]. While labeling was not discussed in that study, the divided views on labeling among our participants, along with the limited policy guidance on this issue, underscore another critical area in need of development.

Where a greater number of relevant policies were available, there was general agreement on key ethical principles, including the need to obtain informed consent, disseminate results, and maintain confidentiality. However, discrepancies emerged regarding who should determine the transfer, storage, and future use of biospecimens – institutions, participants, or research protocols. Consent guidelines also varied with regards to the appropriate age of consent and the type of consent to be used - standard, broad, or separate consent for storage and future use. Given that participants in our study also expressed differing views on biospecimen ownership and the need to re-contact and re-consent for future research, resolving these policy inconsistencies is critical.

## Limitations

There are several limitations associated with this study. First, interview responses were drawn from a narrow geographical region in western Kenya, whereas the reviewed policies spanned a broader region, including countries throughout sub-Saharan Africa. This mismatch limits the comparability between participant perspectives and policy content, and constrains the generalizability of our findings. Second, only a small number of policies (12) were identified, many of which were outdated, making it difficult to draw definitive or actionable conclusions based on their content. Third, there were limitations in how participants were grouped for analysis: YPLHIV with and without prior research participation were analyzed together despite their different experiences, and SMEs were grouped together regardless of their varied professional roles and work settings. Although not explored here, disaggregating these subgroups in future analyses may yield additional insights.

## Conclusion

Perceptions regarding ethical considerations in research involving biospecimens from African YPLHIV demonstrated areas of consensus, though inconsistently, with notable discrepancies between participant views and often limited and outdated policy documents. These findings emphasize the need for clearer, more current, and more comprehensive policy guidance to support ethically sound research involving this vulnerable population. Moving forward, further research should include more nuanced analyses of participant groups to better understand potential differences in perspectives both between and within them. Policymakers should prioritize inclusive, regularly updated guidance that incorporates diverse stakeholder input. When stakeholder views diverge, policies should err on the side of inclusivity and caution, acknowledging and addressing a range of concerns where feasible to protect participants, build trust, and fill identified gaps. Greater standardization across policies may also help promote a more cohesive and ethically grounded framework for research involving specimen biobanking in YPLHIV.

## Supplementary Information

Supplementary Material 1. Table S1: African policy topics related to major interview themes.

## Data Availability

The datasets used and/or analyzed during the current study are available from the corresponding author on reasonable request.

## Abbreviations

AMPATH: Academic Model Providing Access to Healthcare
ART: Antiretroviral Therapy
HIV: Human Immunodeficiency Virus
IRB: Institutional Review Board
IREC: Institutional Research and Ethics Committee
MTRH: Moi Teaching and Referral Hospital
NACOSTI: National Commission for Science, Technology and Innovation
SME: Subject Matter Expert
YPLHIV: Young People Living with HIV
