# Supplementary material for: The ethics of research involving biobanking specimens from Kenyan children and adolescents living with HIV: discrepancies between individual perceptions and policy considerations
Source: BMC Med Ethics. 2026 Feb 26;27:67. doi: 10.1186/s12910-026-01421-7 (PMC13040865; doi:10.1186/s12910-026-01421-7)
Supplement: Supplementary file 1 [file 12910_2026_1421_MOESM1_ESM.docx]

Supplemental Table 1. African policy topics related to major interview themes.

| Policy, Year, and Document Type | Collection and Analysis of Biospecimens | Storage and Identification of Biospecimens | Testing and Sharing of Biospecimens |
| --- | --- | --- | --- |
| Informed Consent for Collection and Use | Disseminating Results to Participants | Delineating Participant Benefits in Study Documentation | Storage and Informed Consent | Confidentiality | Biospecimen Labeling | Future Use and Informed Consent | Biospecimen Sharing |
| Botswana, 2005 (Guideline) [20] | Consent forms must indicate usage. | Consent form should outline procedures for informing participants of results. |  | Consent forms must indicate storage length and location. | Researchers should inform participants of confidentiality procedures. |  | Consent forms must indicate future procedures. | Consent form must list the institutions allowed to review participant data. |
| Ethiopia, 2014 (Guideline)  [21] | The consent form should describe intended use. | Participants will receive general results, not individual results. | Consent forms must outline expected benefits to the participant and the community. | Separate consent is required for storage. | The collection, processing, handling, storage, transfer, and destruction of biospecimens and data should be conducted in a way that protects confidentiality. | Only anonymized specimens should be used for secondary or third-party purposes. | Separate consent is required future use. | Participants have the right to decide to share biospecimens. |
| Kenya, 2017 (Standard Operating Procedure) [22] |  | Consent forms must state how results will be available to participants and their communities. | Consent forms must describe potential benefits to participants and community members. |  | Participants must provide consent for researchers to publish study information. |  | Consent forms should indicate if future use of data beyond the current study is anticipated. | Consent forms must outline the purpose and location of testing for transferred biospecimens. |
| Kenya, 2020 (Guideline)  [23] | Researchers sending biospecimens abroad should ensure consent was obtained during collection. | Participants will receive general results, not individual results. |  | Ethics committee approval should be obtained following participant consent for storage. | Researchers are required to uphold confidentiality by protecting personal information and ensuring data gathered is only utilized or shared for reasons consented to. |  | Additional consent is required for further tests. | Consent forms must indicate that biospecimens will be shared. |
| Malawi, 2007 (Guideline)  [24] | Consent must be given prior to biospecimen collection. | Protocols must include results dissemination plans, especially within Malawi. |  |  | Consent form must outline confidentiality measures along with who is allowed to review participant information. |  |  | Consent forms must outline the purpose for sharing biospecimens. |
| Malawi, 2012 (Guideline)   [25] | Consent form must describe the collection and use of genetic information. | Researchers must have results available, but participants can decide whether or not to receive the results. |  | Consent form must describe storage. | Researchers are required to demonstrate procedures for protecting the confidentiality of genetic data. | Research protocols must include plans for anonymizing genetic materials. |  |  |
| Nigeria, 2013 (Policy Statement) [26] |  |  |  | Stored biospecimens must match consent form. | Personal identifying data should be anonymized. | Specimens must be anonymized. | Broad consent can be used for future use. Study documents must describe biospecimen removal and destruction when future use is declined. |  |
| Rwanda, 2009 (Standard Operating Procedure)  [27] | Consent form is required to be in English and local languages and must describe biospecimen collection. | Researchers must outline procedures for informing participants of results. Participants must be informed of findings used in publications and conferences. |  | Separate consent is required for storage. | Consent form must be used to collect confidential information. |  | Separate consent is required for future use. |  |
| South Africa, 2017 (Guideline)    [28] | Consent form must be completed by participants older than 16 years before biospecimen collection. | Participants have a right to know study results. | Community benefits must be included in the study protocol. |  | Biospecimens must remain confidential. | Clear, accurate labeling for transport and storage is required. | Consent form must be broad enough to allow for secondary uses. | A material transfer agreement is required to share biospecimens, which must be used for a research-related purpose. |
| Sudan, 2008 (Guideline)   [29] |  | Participants have a right to know both individual and overall health results from the study. |  | Participants can decide future use and destruction of biospecimens. Consent form must describe storage length and location. | Privacy measures must be established to ensure confidentiality of identifiable records. |  | Consent form must describe future use and separate consent is required for additional research. While participants can make future use decisions, biospecimens can be used without consent if the ethics committee determines lack of risk. | Participants can make biospecimen transfer decisions. |
| Tanzania, 2023 (Guideline)   [30] |  | Participants have a right to know study results. | Consent forms should describe participant benefits. | Institutions can make storage decisions. | Consent form must describe confidentiality measures and be the only document linked to the participant. |  | Consent form must describe future use beyond original research purposes. Institutions can make usage decisions. | Researchers must adhere to the sharing protocol outlined in the consent form. Institutions can make transfer decisions. |
| Uganda, 2014 (Guideline)   [31] |  | Researchers must share findings with participants, community members, and other stakeholders. |  | Organizations can make storage decisions. Separate consent is required for storage which should include storage quantity and location. | Research must be conducted in a way that protects confidentiality and privacy. Consent form should be the only link to the participant. |  | Separate consent is required for future use, though organizations can make future use decisions. | Organizations can transfer biospecimens on behalf of the participant through a materials transfer agreement. Organizations can make transfer decisions. |
| Zambia, 2013 (Act) [32] | Consent form must be completed before biospecimen collection and use. | Results must be shared locally before sharing abroad. |  |  | Privacy and confidentiality must be maintained. |  |  | Approval must be obtained before sharing biospecimens. |
| Zimbabwe, 2004 (Guideline)   [33] |  | Participants have a right to know study findings. |  | Consent form must allow participants to decide about storage and destruction. | Participant must approve the disclosure of confidential information. |  | Consent form must allow participants to decide about future use. |  |
